# Structural optimization of silicon thin film for thermoelectric materials

**DOI:** 10.1038/s41598-021-01855-6

**Published:** 2021-11-22

**Authors:** Takuma Hori

**Affiliations:** Department of Mechanical Systems Engineering, Tokyo University of Agriculture and Technology, Koganei, Tokyo Japan

**Keywords:** Mechanical engineering, Thermoelectric devices and materials

## Abstract

The method to optimize nanostructures of silicon thin films as thermoelectric materials is developed. The simulated annealing method is utilized for predicting the optimized structure. The mean free path and thermal conductivity of thin films, which are the objective function of optimization, is evaluated by using phonon transport simulations and lattice dynamics calculations. In small systems composed of square lattices, the simulated annealing method successfully predicts optimized structure corroborated by an exhaustive search. This fact indicates that the simulated annealing method is an effective tool for optimizing nanostructured thin films as thermoelectric materials.

## Introduction

Ubiquitous devices will be indispensable for enriching human life in the future. Small, sustainable and maintenance-free energy sources are needed for these ubiquitous devices. One of the candidates is thermoelectric devices that produce electric current by imposing temperature difference. While many types of materials have been suggested, silicon is notable material for thermoelectric power sources of ubiquitous devices since it is less toxic and more abundant than other materials^1^. In fact, thermoelectric power generators composed of silicon nanostructures have been reported^2–4^. However, the limited conversion efficiency of the silicon-based thermoelectric materials at room temperature restricts their practical use. The conversion efficiency of thermoelectric materials is determined by the dimensionless parameter, *ZT* = *S*^2^*σT*/*κ*, where *T* is the absolute temperature, *S* is the Seebeck coefficient, *σ* is the electrical conductivity, and *κ* is the thermal conductivity^5^. The thermal conductivity of semiconductor materials such as silicon is governed by phonon transport. Thus, it is important to reduce the lattice thermal conductivity to achieve higher conversion efficiency and optimize the electrical properties.

Nanostructuring has been a popular approach to improving thermoelectric efficiency in recent decades to promote the phonon-boundary scattering by dense interfaces of nanostructures, resulting in a reduction in thermal conductivity without sacrificing electrical properties^6–9^. Nanostructured silicon thin films fabricated from silicon on insulator are one of the typical ones. This type of nanostructured thermoelectric materials can be directly fabricated on integrated devices. Early studies have shown that the nanoporous silicon thin film exhibits high thermoelectric performance^10^. Subsequently, several studies have demonstrated the fabrication of silicon porous thin films for thermoelectric application and for investigating phononic effect^11–17^. Modulated nanowires, which are similar structures with porous films, have also been fabricated from silicon on insulators^18–23^. These nanostructured silicon thin films have exhibited notable thermal conductivity reduction. To realize further optimization, theoretical or simulational support is necessary since the fabrication of these nanostructures is costly.

Numerical approaches have been used to optimize nanostructures as thermoelectric materials since thermal conductivity prediction is available before the fabrication. Harmonic and an harmonic lattice dynamics^24–28^ can evaluate intrinsic properties of phonons such as dispersion relation and scattering rate. The thermal conductivity of arbitral nanostructures can be assessed by combining phonon properties and the Boltzmann transport equation for phonon transport, in particular, under the relaxation time approximation^29^. Most of the studies use stochastic approaches including the Monte Carlo simulation^30–34^ to solve the Boltzmann transport equation since they are suitable for dealing with complex boundary conditions reflecting the nanostructures.

Many numerical studies have focused on predicting the thermal conductivity of silicon nanostructures, including silicon porous thin films^13,35–38^ and modulated nanowires^21,39,40^. In these studies, the influence of the characteristic length of nanostructures, such as hole size, neck length, and film thickness, on the thermal conductivity has been revealed. However, the best nanostructures fabricated from silicon on insulators for thermoelectric materials remain unknown. Hence, it is necessary to identify the structures of the lowest thermal conductivity for thermoelectric application. Although the method for predicting optimized structure in atomistic is already proposed^41^, that applicable to the larger scale ones has not been reported. In this study, the thermal conductivity of nanostructures fabricated from silicon on insulator substrate is investigated by establishing a framework to search the optimized structure for thermoelectric materials.

## Method

**Figure 1:**
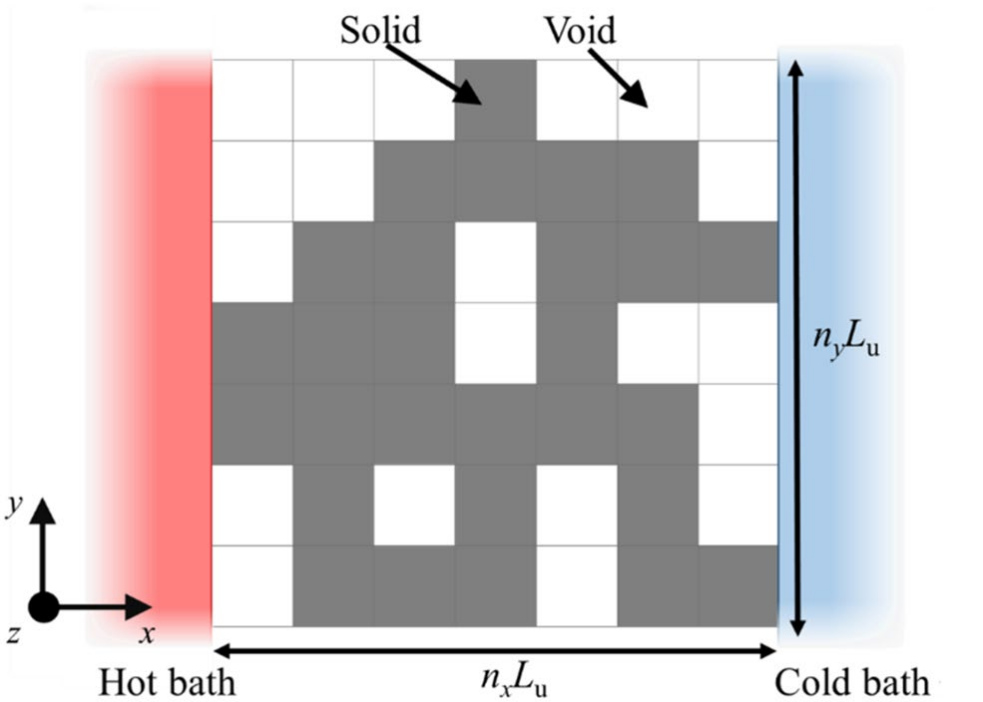
Schematic diagram of a nanostructured thin film. The film connected to hot and cold bathes is composed of square lattices whose side length is *L*_u_. The number of the lattices is *n*_*x*_ for *x*-direction, and *n*_*y*_ for *y*-direction. The film thickness is *L*_z_.

Figure 1 shows the geometry of nanostructured thin film investigated by numerical approaches. Each edge of the film of uniform thickness *L*_*z*_ is connected to a hot and cold bath, respectively. The numerical film model is discretized into square lattices of side length *L*_u_. These length parameters are set as *L*_*z*_ = *L*_u_ = 100 nm. Each lattice is assigned as solid or void to mimic complex nanostructures; for example, if all the lattices are solid, the structure is identical to a plain thin film with finite length and width. The number of those lattices are *n*_*x*_ and *n*_*y*_ along *x*- and *y*-direction, respectively. Therefore, the total number of nanostructure patterns reproduced by these lattices is 2^*N*^ where *N* = *n*_*x*_ × *n*_*y*_.

The thermoelectric performance can be characterized by *ZT* = *S*^2^*σT*/*κ*. Among these physical properties, Seebeck coefficient *S* is determined by electrical band structure. If the characteristic length of nanostructures is longer enough than the wavelength of electrons in crystals, it remains unchanged. Besides, the assumption of constant electrical conductivity is supported by the difference of mean free path of phonons and electrons; the former is much larger than in silicon at room temperature^42^. Hence, the electron-boundary scattering can be neglected, and the electrical conductivity becomes insensitive to the nanostructure. Meanwhile the phonons frequently scatter with boundaries since the mean free path of phonons ranges from 10 nm to 10 μm at room temperature^25^, resulting in thermal conductivity reduction. Therefore, the thermal conductivity of silicon thin film whose characteristic length is 100 μm is only the essential property for its thermoelectric conversion efficiency, as also suggested in the previous^10^.

The thermal conductivity of the nanostructured thin film can be obtained using the phonon gas model. In this model, the thermal conductivity suppressed by phonon-boundary scattering *κ*_eff_ can be expressed as follows:

1 $$\kappa_{{{\text{eff}}}} = \frac{1}{3}\sum\limits_{{{\mathbf{k}}s}} {C_{{{\mathbf{k}}s}} v_{{{\mathbf{k}}s}} \Lambda_{{{\mathbf{k}}s{\text{, eff}}}} } ,$$

where *C*, *v*, and Λ are the specific heat, group velocity, and mean free path, respectively. The subscripts **k** and *s* indicate wave vector and branch. Here the effective mean free path Λ_**ks**, eff_ can be calculated from the mean free path determined by material, Λ_**ks**_, and that by structure, Λ_bdy_,

2 $$\Lambda_{{{\mathbf{k}}s,{\text{eff}}}}^{ - 1} = \Lambda_{{{\mathbf{k}}s}}^{ - 1} + \Lambda_{{{\text{bdy}}}}^{ - 1} .$$

More specifically, the first and second terms of the right hand side of Eq. (2) are originated from phonon–phonon and phonon-boundary scatterings, respectively. Other scattering mechanisms are not included. Among these properties, specific heat, group velocity, and mean free path determined by the material are obtained by lattice dynamics^29^ with interatomic force constants based on first principles. In this study, the phonon properties in bulk silicon crystals at room temperature are calculated by using ALAMODE^26^. Here, 30 × 30 × 30 mesh points in reciprocal space are utilized for the lattice dynamics calculation. The calculated thermal conductivity of the bulk crystal is about 150 Wm^−1^ K^−1^ at room temperature, consistent with the experimental value^43^. More details on the calculations of lattice dynamics can be found in the previous study^44^, where the same calculations were carried out.

Meanwhile, the mean free path due to structure, Λ_bdy_, which is the only parameter determined by the structure, is calculated by phonon transport simulations. Here, the ray tracing method is employed^44,45^. This method is applicable to analysis of phonon transport under small temperature difference. Compared with the traditional Monte Carlo simulations often utilized to obtain thermal conductivity of nanostructures, the ray tracing method cannot simulate transient phenomena but needs much less computational cost, which is beneficial for optimizing the structures since the evaluation of the properties of many structures are necessary. It has been widely utilized for analyzing phonon transport in various structures^13,14,21,35,37,45–48^, and is also known as the test particle method in the field of rarefied gas dynamics^49,50^. Note that the validity of the method is ensured in the previous studies where the agreement between the mean free path of a plain thin film^13^ and nanowire^45^ calculated by the method and that by theories is confirmed. In this method, the phonon transport emitted from one side of the heat bath is simulated until they return to the inlet edge or reach the opposite one. During the migration, phonons collide with boundaries of nanostructures without intrinsic scattering; this collision is assumed to be diffuse scattering, where the direction of the colliding phonons is randomly reassigned. The transmission probability $\mathcal{T}$ is obtained by introducing many phonons repeatedly. Finally, Λ_bdy_ for each structure can be evaluated by the following equation as shown in the previous study^44^,

3 $$\Lambda_{{{\text{bdy}}}} = \frac{3\mathcal{T} L}{{4f}},$$

where *L* and *f* refer to the length of the structure (= *n*_*x*_*L*_u_), and the correction factor which represents non-uniformity of the cross-sectional area of the structure, respectively. In this study, 10,000,000 or 50,000,000 phonons are simulated for each nanostructure depending on their size. The finite volume method to solve the heat conduction equation is employed to determine *f*; it is obtained by evaluating the ratio of the heat flux of the structures at the inlet boundary and that under assuming constant-cross sectional area^44^. Orthogonal mesh whose size is *L*_u_/20 is used in the finite volume method.

## Results and discussion

**Figure 2:**
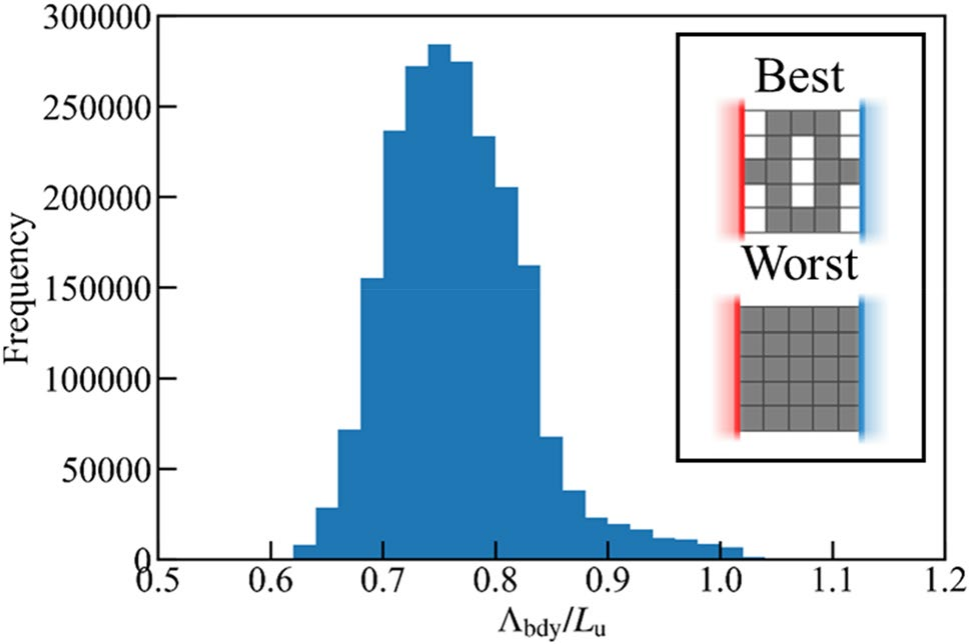
Histogram of Λ_bdy_ in lattice structures of *n*_*x*_ = *n*_*y*_ = 5 calculated by Eq. (3). Λ_bdy_ is normalized by the unit length of lattice *L*_u_. Inset shows structures whose Λ_bdy_ is minimum and maximum.

To specify the optimized nanostructure of silicon film as a thermoelectric material, Λ_bdy_ of all the patterns represented by the lattice structures illustrated in Fig. 1 is evaluated. Owing to the computational cost, phonon transport in the structure with *n*_*x*_ = *n*_*y*_ = 5 is simulated; even in this extremely small system, the number of the patterns of the nanostructures represented by solid and void lattices is enormous (2^25^ = 33,554,432). However, the number is reduced to ten times smaller by ignoring unrealistic structures. For example, if there are isolated solid lattices in the film, it is impossible to support them thus the structure cannot be realized. Besides, if the structure is disconnected from either of the heat baths, it is meaningless since there is no heat and electric current. These structures are excluded before calculating Λ_bdy_ by numerically labelling solid lattices whether they are connected to neighbor ones and the heat bathes. Excluding these unrealistic structures, the number of the structures constructed by the lattices is reduced to 2,140,215. The frequency of Λ_bdy_ calculated by Eq. (3) is shown in Fig. 2. It is found that for almost all the structures, Λ_bdy_ is about 0.75*L*_u_. The structure of the longest Λ_bdy_ is a plain thin film with Λ_bdy_/*L*_u_ = 1.15, whereas that of the shortest one is 0.59, showing that the shortest one is around half times smaller than that of the plain one.

**Figure 3:**
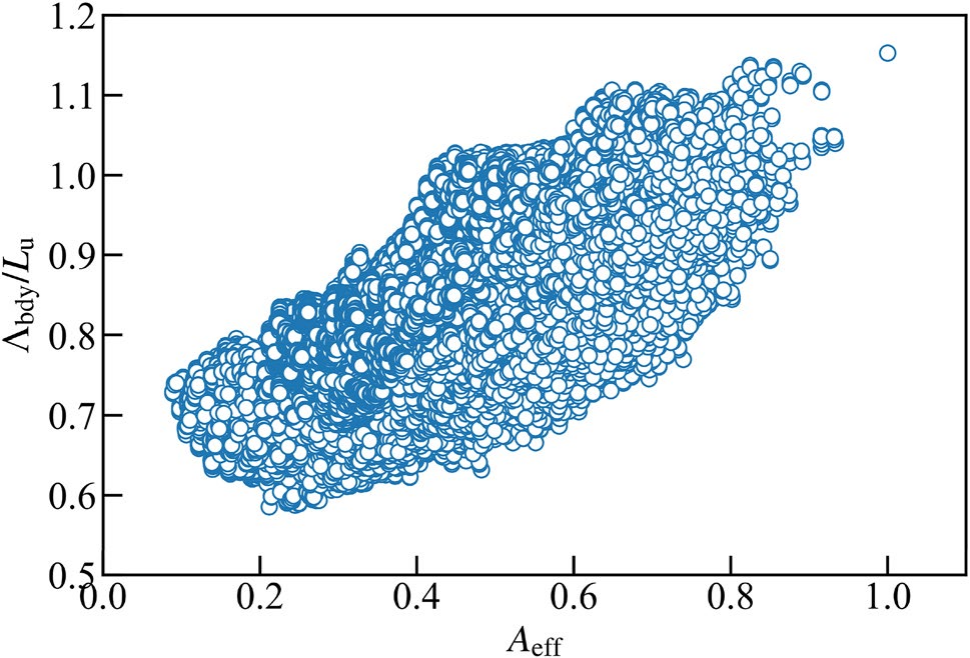
Correlation between the effective area for diffusive transport *A*_eff_ and mean free path of boundary scattering Λ_bdy_/*L*_u_.

The simulations can also predict the more practical design of thermoelectric materials. While nanostructuring can reduce the thermal conductivity, the amount of the electric current through the structure must be ensured at the same time. In other words, if the cross-sectional area is reduced by nanostructuring, the generated power from thermoelectric devices is limited; it is not useful even if the conversion efficiency is high. Therefore, the effective cross-sectional area for diffusive transport is another crucial factor for thermoelectric performance. Here the effective cross-sectional area *A*_eff_ can be evaluated as *A*_eff_ = *fA*_in_ where *A*_in_ is the cross sectional area at the inlet boundary normalized by that of the plain thin film *n*_*x*_*L*_u_^2^. For example, *A*_eff_ of plain thin film and the square nanowire of side length *L*_u_ in *n*_*x*_ = *n*_*y*_ = 5 system are equal to 1 and 0.2, respectively. Obviously, the former one is better than the latter one in terms of the heat current path. Figure 3 summarizes the correlation between *A*_eff_ and Λ_bdy_ of all the structures. As can be seen from Fig. 3, *A*_eff_ and Λ_bdy_ have a positive correlation. This result is reasonable since the nanostructuring induces an increase in phonon-boundary scattering and a decrease in thermal conduction path. However, *A*_eff_ of the structure whose Λ_bdy_ is the smallest of around 0.2, is not the worst one. This result indicates that while the structures are optimized for conversion efficiency, there is still room to choose better structures in terms of power generation.

The results of very small cases have been simulated owing to the high computational cost of exhaustive search; only five lattices are arranged for *x*- and *y*-direction. Meanwhile, the calculation of much larger systems should be necessary for application. To overcome the problem of computational cost, simulated annealing method is utilized. In the simulated annealing method, the structures are gradually deformed in each step, and the better ones are survived for next steps. The detail of the method is explained as the followings. The method iteratively calculates and minimizes the objective function *E*, which corresponds to Λ_bdy_ of the various nanostructures in this study. In every international step, the new structure is generated by randomly choosing *N*_c_ lattices of the former structure and switching their solid/void states. Therefore, only the neighboring state of the local minimum solution is simulated, not like the exhaustive search. The structures not connecting to both heat baths are excluded during the generation of the new structures. Besides, isolated lattices in nanostructured films are ignored. Then Λ_bdy_ is calculated and the new structure is accepted if the objective function of the new structure *E*_new_ is smaller than the current one *E*_cur_. Even for *E*_new_ > *E*_cur_, the transition from the current state is accepted depending on the probability *P* = exp(− Δ*E*/*E*_T_), where Δ*E* and *E*_T_ are the normalized difference of the objective functions (*E*_new_ /*E*_cur_ −1) and a time-varying parameter often called “temperature” in the simulated annealing method, to avoid falling into a locally optimal solution. For *E*_T_ = zero, the method is called the hill-climbing method. On the other hand, the method is equivalent to random search when *E*_T_ is infinity. At the same time, *A*_eff_ is calculated to use as a threshold of acceptance; even when *E*_new_ satisfies the above transition conditions, it is declined if *A*_eff_ is smaller than a preset threshold *A*_eff,thr_.

**Figure 4:**
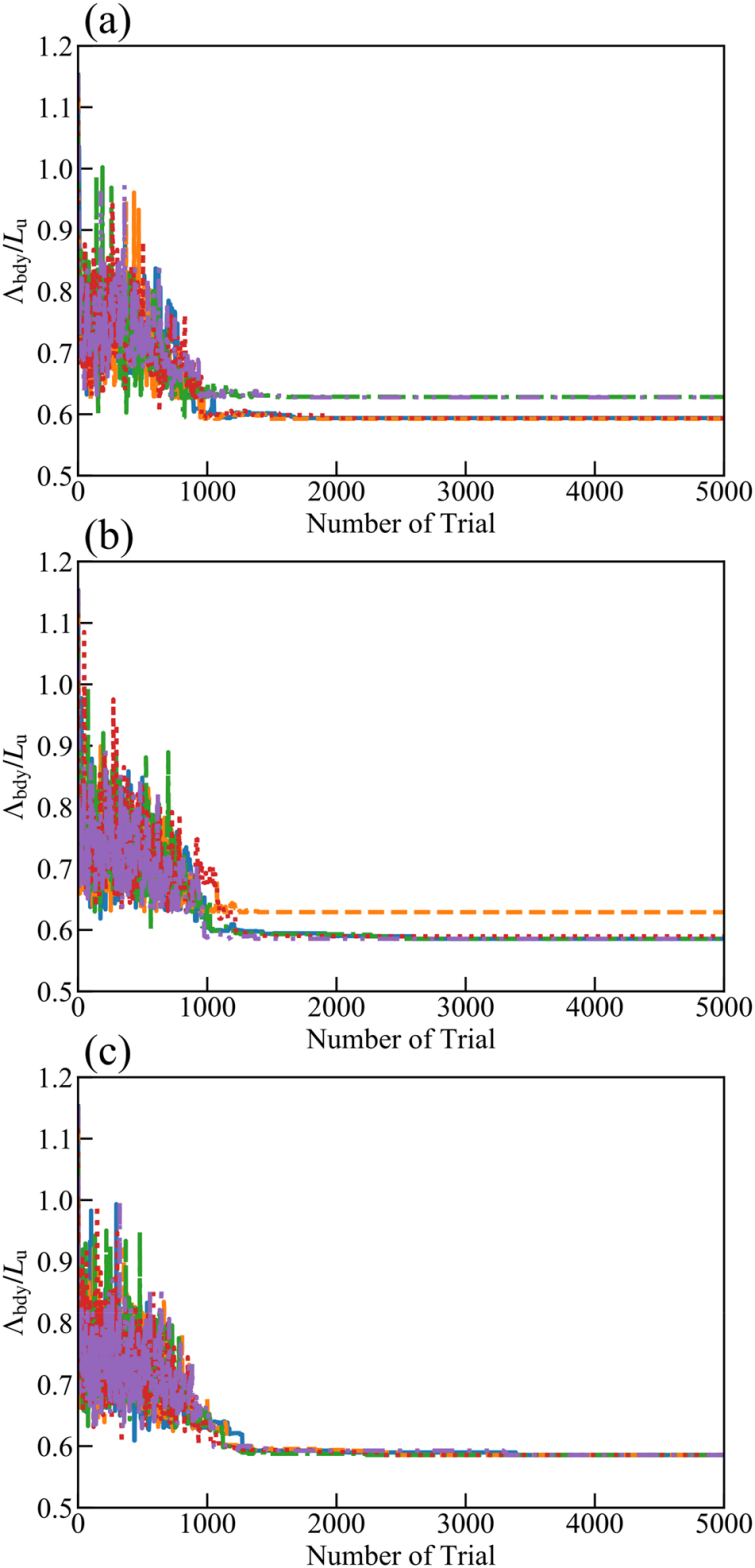
Evolution process of Λ_bdy_ /*L*_u_ during an iteration of simulated annealing method where *N*_cmax_ = (**a**) 1, (**b**) 3, and (**c**) 5. Five lines show the results for the same size of thin film (*n*_*x*_ = *n*_*y*_ = 5) with different random seeds.

Parameters in the methods should be chosen to use the simulated annealing method to predict the optimized structure with a small computational cost. The impact of the parameters on the evolution of the objective function is assessed in the simulated annealing method. First, the way to search neighboring states is discussed. Figure 4a–c shows a trial number of the switching of solid void states and the updates of Λ_bdy_. Here, the parameter *N*_c_ is uniformly chosen from 1 to *N*_cmax_; for the insets (a–c), *N*_cmax_ is set to 1, 3, and 5. The temperature *E*_T_ is initially set to be unity, and multiplied by 0.8 every *N*_temp_ = 50 steps, and *A*_eff,thr_ is fixed to 0. The initial states are identical to the plain thin films. The results with five different random seeds are shown in each figure. When *N*_cmax_ is 1 or 3, some of the simulations cannot reach the optimized state where Λ_bdy_/*L*_u_ is 0.59 as predicted by the exhaustive search shown in Fig. 2. In contrast, all Λ_bdy_ converge to the minimum value when *N*_cmax_ is 5 since simultaneous switching of the states of many lattices promotes the evacuation from local minimum states. Meanwhile, a much larger *N*_cmax_ decelerates the convergence process. Hence, *N*_cmax_ is fixed to 5 for the following simulations.

**Figure 5:**
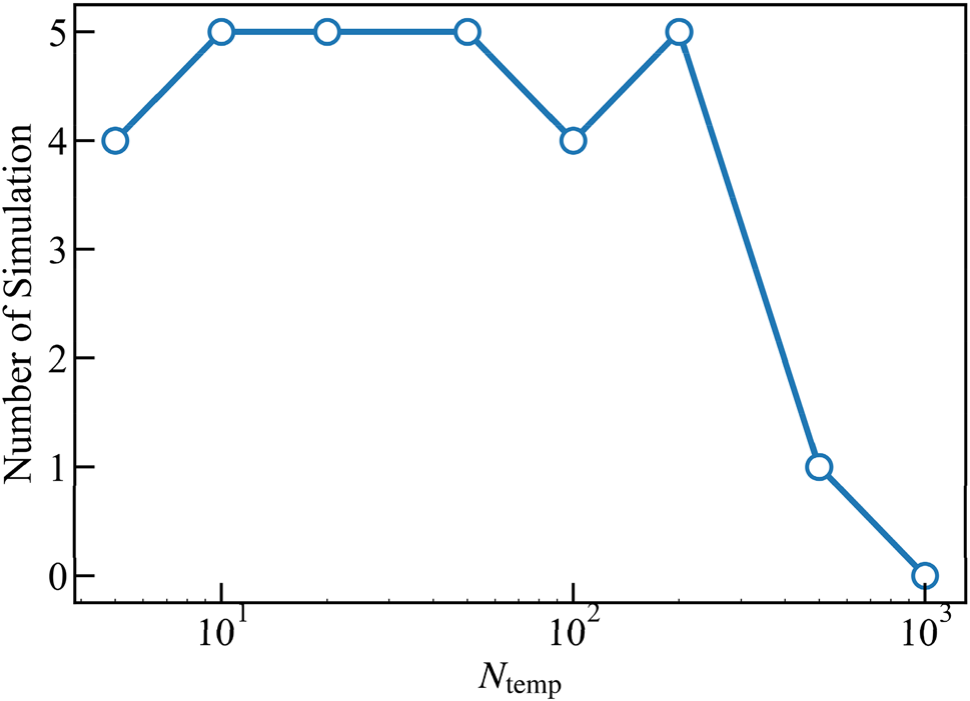
Number of simulations successfully predict the local minimum state out of five different ones within 10,000 trials. *N*_temp_ is the time step to decrease temperature.

The effect of the temperature set in the simulated annealing method on its prediction is also evaluated. The rate of temperature decrement *N*_temp_ varies from 5 to 1000 while the initial temperature is fixed to 1. The smaller *N*_temp_ implies the temperature decrease is fast, which leads to *P* = 0; thus, the iteration process is close to the hill-climbing method, which does not incorporate probabilistic criteria for accepting a new structure. On the other hand, the larger *N*_temp_ results in random choice, which is not suitable for computational cost. Figure 5 presents the number of simulations within 10,000 trials that successfully predict the local minimum state out of five different ones. When *N*_temp_ is large, the simulations fail to reach the optimized structure. Considering that the smaller *N*_temp_ leads to slow convergence, *N*_temp_ is fixed to 50.

**Figure 6:**
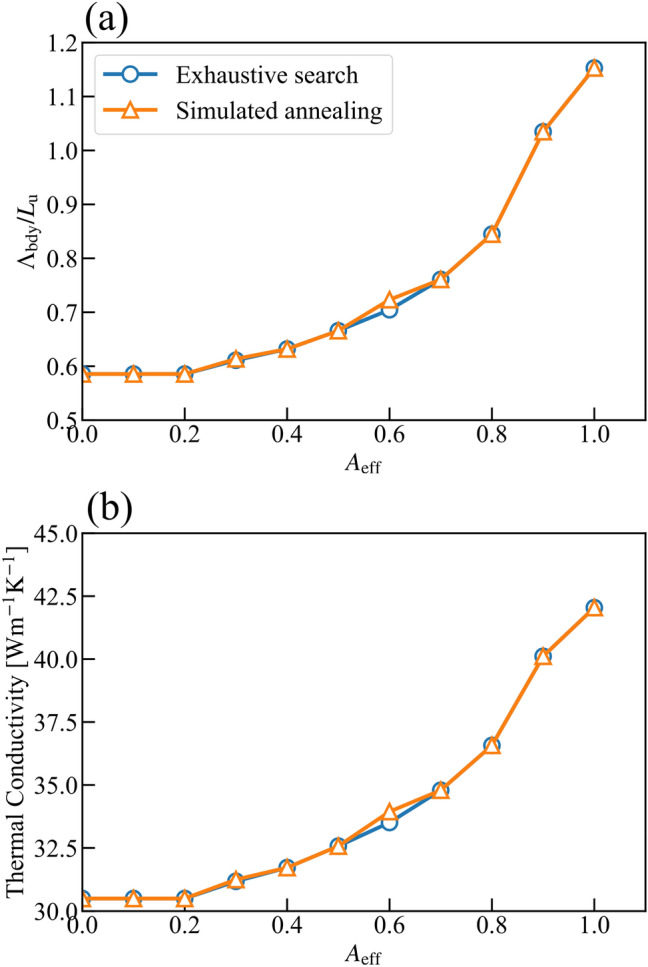
Relation between *A*_eff_ and (**a**) Λ_bdy_ or (**b**) thermal conductivity of nanostructured silicon thin film at room temperature. Thermal conductivity is calculated by Eqs. (1) and (2). A comparison between the results of the exhaustive search and the simulated annealing is shown.

The results are compared with those of an exhaustive search to quantitatively evaluate the validity of the simulated annealing method. The threshold for effective cross-sectional area *A*_eff,thr_ is set in the simulated annealing method. Again, five independent simulations with different random seeds are performed for the simulated annealing method with each condition, and the best results are plotted in Fig. 6a. Most of the results of an exhaustive search shown in Fig. 3 are omitted, and only Pareto optimal solutions at each *A*_eff_ among them are plotted for comparison, as shown in Fig. 6a. There is a good agreement between those two methods. The former and latter ones investigate 2,140,215 and 50,000 (10,000 for five simulations) candidates, respectively. Thus, the heuristic approach, whose computational cost is much lower than the brutal search, is an efficient tool for optimizing the thermoelectric performance of nanostructures, and is also appropriate for a much larger simulation system.

The thermal conductivity is also evaluated by substituting Λ_bdy_ shown in Fig. 6a and other properties such as group velocity into Eqs. (1) and (2) to investigate the quantitative effect of nanostructuring on *ZT*. The minimum thermal conductivity among the structures whose *A*_eff_ is greater than the threshold value *A*_eff, thr_ is plotted in Fig. 6b. The results show that the thermal conductivity can be decreased by more than 10 Wm^−1^ K^−1^ via nanostructuring. However, the effective area is also reduced to 20%. In this way, the simulations can provide the balance between the conversion efficiency and the amount of current produced by the device.

**Figure 7:**
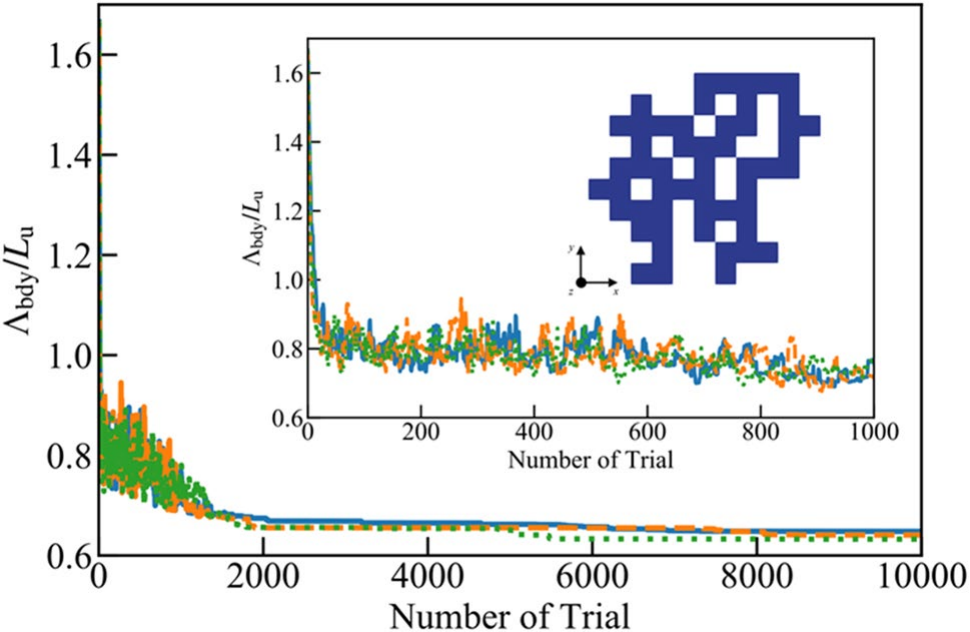
Evolution process of Λ_bdy_/*L*_u_ during an iteration of simulated annealing method for large thin film (*n*_*x*_ = *n*_*y*_ = 11). Three lines indicate the results with different random seeds. The inset shows an enlarged view of the small trial number area. One of the predicted structures is also shown.

The simulated annealing method is used for the larger one, where *n*_*x*_ and *n*_*y*_ are 11. This is achieved by parallelizing both the ray-tracing simulation and finite volume method simulation; while the former does not need to be explicitly parallelized since it evaluates the probability of phonons transmission through structures, the latter one is parallelized by dividing the simulation domain. In total, 5,000,000 particles are simulated in the ray-tracing method. The thickness of the film *L*_*z*_ is the same as *L*_u_ as in the previous simulations. The parameters of the simulated annealing method are set to *N*_max_ = 5 and *N*_temp_ = 50 consulting the smaller systems, and 10,000 trials are performed for each simulation. Here, three independent simulations starting from plain thin films with a different random number are performed. Again, during optimization, Λ_bdy_ is gradually updated by the simulated annealing method, as shown in Fig. 7. The results reveal that Λ_bdy_ of the three simulations has been improved compared to the initial plain thin film (Λ_bdy_ = 1.67*L*_u_) to a range between 0.63 and 0.65*L*_u_. One of the examples is shown in Fig. 7. Then Λ_bdy_ of typical structures are also evaluated for comparison. For example, the porous structure where 16 void lattices (out of 121 lattices) are orderly aligned shows Λ_bdy_ = 0.89*L*_u_; when the void lattices of same number are placed in staggered alignment, Λ_bdy_ exhibits 0.79*L*_u_. These values of typical porous structures are much larger than that of the optimized structures predicted by the simulated annealing method, although there is no guarantee that the predicted one is the “absolute optimal configuration”. In addition, Λ_bdy_ of the structures found by three different simulations are almost same values, suggesting that it is close to the lower limit. This fact indicates that the simulated annealing method can predict the better structure even in large systems, although there is still a possibility that they are local minimum solutions. Further optimization of the parameters and a more sophisticated heuristic method are needed to accurately and rapidly optimize thermoelectric nanostructures.

## Conclusions

The optimized nanostructures of silicon thin films as thermoelectric materials are numerically investigated by using the simulated annealing method. Phonon transport simulations are employed to evaluate the mean free path of phonons in nanostructured thin films represented by square lattices. The mean free path of all the structures represented by 25 lattices is also calculated for comparison. As a result, the simulated annealing method successfully predicts the optimized structure found by the exhaustive search. This agreement ensures the validity of the method for searching the optimized nanostructure. The thermal conductivity reduced by nanostructuring is also evaluated with using phonon properties calculated by lattice dynamics with interatomic force constants obtained from the first principle calculations. The thermal conductivity of the optimized structure is found to be about 0.7 times smaller than that of the plain thin film. Finally, the optimized structure of a much larger system composed of 121 lattices where the exhaustive search cannot be carried out owing to the computational cost is investigated by the simulated annealing method. The mean free path of the optimized structures predicted by three independent simulations almost agree with each other, indicating that the structure can be successfully optimized by the method even in larger system. Further improvement of the settings such as initial condition, local search, and avoidance of local minimum would help us to access the best structure as thermoelectric materials even in larger and more complex systems. For example, the method can be applied to optimize surface roughness of nanowires, grain size distribution of sintered polycrystals, and three-dimensional porous structures.
